# Coexistence of Trichome Variation in a Natural Plant Population: A Combined Study Using Ecological and Candidate Gene Approaches

**DOI:** 10.1371/journal.pone.0022184

**Published:** 2011-07-19

**Authors:** Tetsuhiro Kawagoe, Kentaro K. Shimizu, Tetsuji Kakutani, Hiroshi Kudoh

**Affiliations:** 1 Department of Biology, Faculty of Science, Kobe University, Kobe, Japan; 2 Institute of Plant Biology, University of Zurich, Zurich, Switzerland; 3 National Institute of Genetics, Mishima, Shizuoka, Japan; 4 Center for Ecological Research, Kyoto University, Otsu, Japan; Centre National de la Recherche Scientifique, France

## Abstract

The coexistence of distinct phenotypes within populations has long been investigated in evolutionary ecology. Recent studies have identified the genetic basis of distinct phenotypes, but it is poorly understood how the variation in candidate loci is maintained in natural environments. In this study, we examined fitness consequences and genetic basis of variation in trichome production in a natural population of *Arabidopsis halleri* subsp. *gemmifera*. Half of the individuals in the study population produced trichomes while the other half were glabrous, and the leaf beetle *Phaedon brassicae* imposed intensive damage to both phenotypes. The fitness of hairy and glabrous plants showed no significant differences in the field during two years. A similar result was obtained when sibling hairy and glabrous plants were transplanted at the same field site, whereas a fitness cost of trichome production was detected under a weak herbivory condition. Thus, equivalent fitness of hairy and glabrous plants under natural herbivory allows their coexistence in the contemporary population. The pattern of polymorphism of the candidate trichome gene *GLABROUS1* (*GL1*) showed no evidence of long-term maintenance of trichome variation within the population. Although balancing selection under fluctuating biotic environments is often proposed to explain the maintenance of defense variation, the lack of clear evidence of balancing selection in the study population suggests that other factors such as gene flow and neutral process may have played relatively large roles in shaping trichome variation at least for the single population level.

## Introduction

Understanding how distinct phenotypes have emerged and how the associated genetic polymorphism is maintained in a natural population or in a species is of great importance in ecology and evolutionary biology [1]. Recent advances in molecular genetic tools have accelerated the exploration of the genetic basis of natural polymorphism in functional traits [2]–[6]. Natural polymorphism in defense traits within single populations has also been found in various plant species [7]–[10]. On one hand, costs and benefits of defense variation have been examined in the contemporary populations to elucidate the ecological mechanism that allows the maintenance of defense variation [11], [12]. On the other hand, recent molecular studies have begun to reveal the genetic basis of defense variation in model species [7], [13]–[15]. However, identifying candidate genes involved in ecologically relevant phenotypic variation remains a challenging task for wild species. Furthermore, the ecological function of polymorphic genes in natural conditions is poorly understood [16]–[19]. In particular, little is known about how genetic polymorphism is maintained within a natural population, because most of relevant studies took a species-wide sampling strategy [2], [4], [20]. As each natural population has unique history of selection and demography, investigating how genetic polymorphism is maintained at the single population level is the first step towards understanding evolutionary processes that have shaped genetic and phenotypic variation.

**Figure 1:**
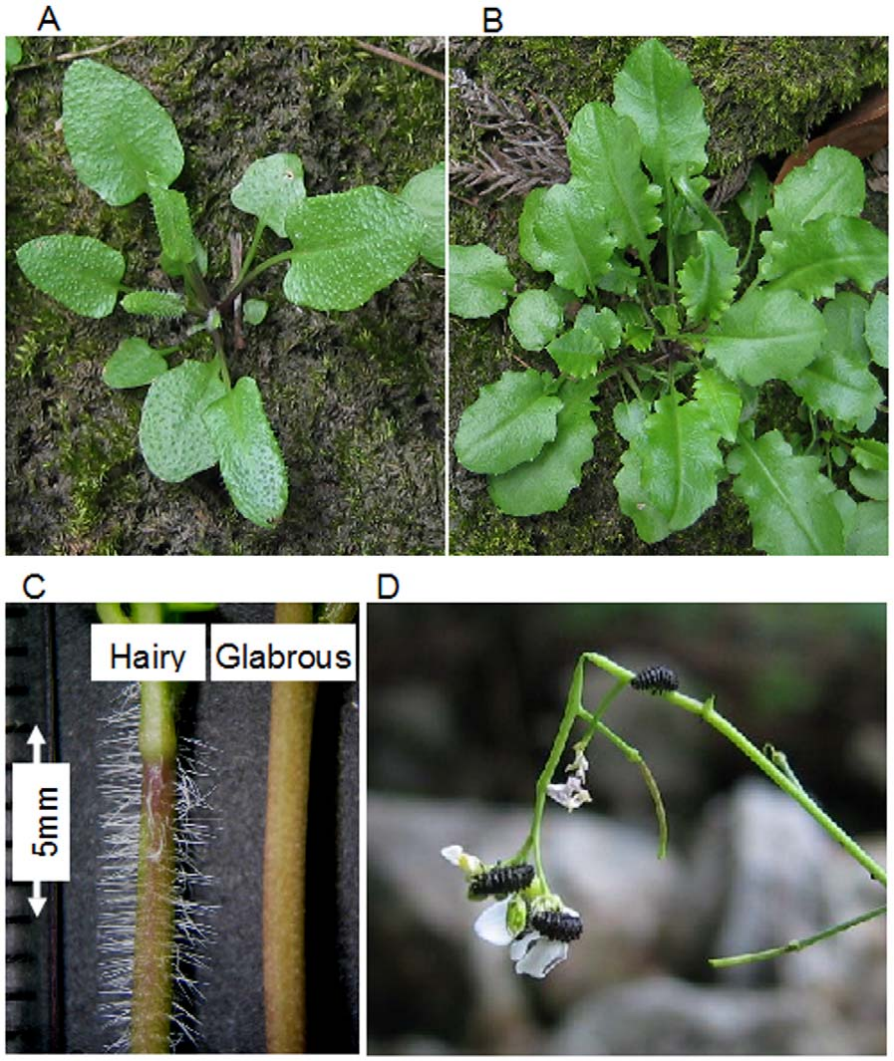
Trichome variation in a natural population of *A. halleri* subsp. *gemmifera*. (A) Hairy plant. (B) Glabrous plant. (C) Flowering stems of a hairy and a glabrous plants. (D) *Phaedon brassicae* larvae foraging on flowers and flower buds.

In the present study, we examined fitness consequences and genetic basis of within-population variation in trichome production in *Arabidopsis halleri* subsp. *gemmifera* (Brassicaceae). In one natural population in Japan, nearly half of the plants develop trichomes on the surface of their leaves and flowering stems (referred to as hairy plants, Figure 1), whereas the other half completely lack trichomes (referred to as glabrous plants). This system provides a unique opportunity to study the genetic basis and ecological consequences of distinct morphological variation for the following reasons. First, previous studies in various species have shown that trichomes represent a defense trait against insect herbivores [21]–[23], including in *A. thaliana* [24], [25], *A. lyrata* [26]–[29] and other Brassicaceae species [30]. However, the detailed knowledge of ecological factors that allow the coexistence of distinct trichome phenotypes within a population is still limited. In particular, relative fitness for different phenotypes needs to be examined across multiple environmental conditions. Second, we are able to adopt a candidate gene approach because *A. halleri* subsp. *gemmifera* is a close relative of the model plant species *A. thaliana* [31], [32]. The molecular genetics of trichome development in *A. thaliana* is well understood, and genes involved in trichome production have been identified [33], [34]. As a candidate gene, we focused on a homologue of *GLABROUS1* (*GL1*), which encodes a MYB-family transcription factor involved in the initiation of trichome development in *A. thaliana* [35]. *GL1* has been found to be responsible for the glabrous phenotype in another population of *A. halleri* subsp. *gemmifera* [36]. *GL1* polymorphisms have also been shown to be associated with trichome variation in natural populations of *A. thaliana* and *A. lyrata* [28], [37]. Furthermore, while most of trichome genes show pleiotropic effects on root hair development, *GL1* is involved in the development of trichomes but not of root hair [33], [34]. Thus, *GL1* is the most promising candidate for trichome variation in *Arabidopsis* relatives.

In this study, we examined the female fitness of hairy and glabrous plants under contrasting herbivory regimes in a natural population. We also analyzed the pattern of *GL1* sequence polymorphism to investigate the association between genotype and phenotype.

## Materials and Methods

### Study system

*Arabidopsis halleri* subsp. *gemmifera* is a perennial herb distributed in East Asia and the Russian Far East [38]. This species is often found in soils contaminated with heavy metals [39]. The study population is also located in an abandoned mine in Hyogo prefecture in the western part of Japan (35°10′N, 134°93′E, ca. 200 m in altitude), where thousands of plants grew along a small creek running through an open forest. Hairy and glabrous plants coexisted in a spatially intermingled fashion, and microhabitat differentiation between the two phenotypes was not found. Hairy plants develop trichomes on the surface of their leaves and flowering stems, whereas glabrous plants lack trichomes (Figure 1A, B, and C). The visible feature of the trichome polymorphism facilitates the ecological study that investigates fitness difference between the distinct phenotypes in natural environments.

Of several insect species that attacked *A. halleri* subsp. *gemmifera* at the study site, the most influential herbivore was the crucifer-specialist leaf beetle *Phaedon brassicae* (Figure 1D). Both adults and larvae of this species fed on leaves and young inflorescences, and larvae were much more abundant than adults in the flowering season [40]. Other herbivorous insects, such as a specialist butterfly, *Pieris napi*, were much less abundant than *P. brassicae*, and their effects on the plants were negligible at the study site (see below).

### Field census

**Figure 2:**
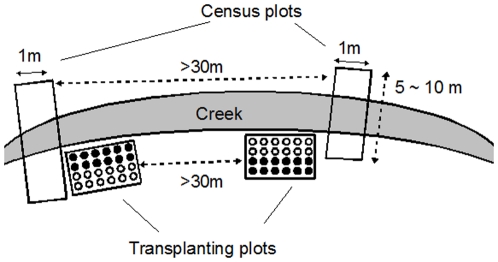
The arrangements of census and transplanting plot. Four census and four transplanting plots were established along a creek. Census plots were established perpendicular to the creek along which plants grew. Plants within the plots were individually marked for regular censuses of herbivory and fitness. A transplanting plot consisted of 12 control and 12 insecticide-treated plants, represented by filled and open circles, respectively. The distance between the two nearest plants was 10 cm.

We conducted a field census for two years (2005 and 2006) to examine differences in the degree of herbivory and fruit production between hairy and glabrous plants. We established four rectangular plots, referred to as ‘census plots’, along the creek (Figure 2, where two of these are shown schematically). The width of a plot was 1 m; the length ranged from 5 to 10 m depending on the distribution of plants; and the distance between nearest plots was greater than 30 m. Almost all plants within the census plots were individually tagged each year, but plants that formed highly dense patches were excluded because the discrimination of individuals was difficult. After tagging, the rosette diameter was measured for each plant prior to the flowering season in 2006 to take plant size into account in our statistical analysis of fitness differences between hairy and glabrous plants. We used 202 hairy and 262 glabrous plants in 2005 and 160 hairy and 199 glabrous plants in 2006 for analysis.

The number of herbivorous insects on each plant was counted once a week on sunny days during the flowering season, which represents the period in the year when herbivory by leaf beetle larvae was most intensive. The damage on leaves was estimated by eye and categorized into one of four levels: 0, no damage; 1, <10% of the total possible area of leaves consumed; 2, <50% possible leaf area consumed; 3, ≥50% possible leaf area consumed. The presence or absence of damage on the apical meristem was also recorded. We adopted this estimate of damage because the rapid loss of leaf tissues due to intensive herbivory did not allow a quantitative measurement to be made.

Fruit production was determined at the end of the flowering season. We did not examine seed production because seeds were spontaneously released from mature fruits, and it was difficult to prevent seed release from fruits while allowing herbivores access to flowers and young fruits. Seed production and male fitness were likely to be strongly correlated with fruit production in the study site because *P*. *brassicae* consumed flowers and flower buds, resulting in the simultaneous loss of both male and female reproductive organs.

We also examined the abundance of herbivores once or twice per month during the summer through winter seasons and found that the intensity of herbivory was weak and negligible. Thus, we present the results only for spring herbivory by *P. brassicae* larvae in this report.

### Insect-removal experiment

To examine the costs and benefits of trichome production, we conducted field transplant experiments in which the intensity of herbivory was manipulated using insecticide (Figure 2). *A*. *halleri* subsp. *gemmifera* reproduces in spring and grows vegetatively in the rest of the year, and *P*. *brassicae* was active from spring to autumn. Thus, two experiments were carried out to test the influences of spring and autumn herbivory. Briefly, the first experiment tested for the influence of autumn and spring herbivory, and the second experiment was aimed at the effect of autumn herbivory on plant fitness. The procedures of the two experiments were mostly same, but differed in the genetic composition of experimental plants (see below).

Seeds for both experiments were collected from the study population in June 2006, and they were germinated under insect-free conditions in the laboratory. The maternal plants from which seeds were collected were at least 10 m apart from each other, and the most distant maternal plants were separated by 300 m. As primary seed dispersal depends on gravity, the probability of sampling related individuals was minimized. For the first experiment, two hairy and two glabrous young plants were randomly selected for each of 24 maternal half-sib families in which the trichome variation was segregated. In total, 96 plants (2 trichome phenotypes×2 plants×24 half-sib families) were used in the first experiment. Seedlings were transplanted individually into a plastic pot (7.5 cm in diameter and 9 cm in depth) filled with vermiculite and grown in a growth chamber (Nihon-Ika, Japan) under conditions of 25°C/15°C with a light regime of 16 h light/8 h dark. The light intensity in the chamber was ca. 200 µmol m^−2^s^−1^ at the pot surface level. For each half-sib family, one hairy and one glabrous plant were assigned to the insecticide treatment, and the others were assigned to the control (no insecticide treatment).

After two months of growth in the laboratory, the plants were measured for rosette diameter, and then pots were placed into four experimental plots (referred to as ‘transplanting plots’) in the field on 3 October 2006 (Figure 2, two of these are shown schematically). Each transplanting plot consisted of 24 plants arranged in a 4×6 grid pattern with 10 cm intervals between the nearest plants (Figure 2). A pot with an individual plant was directly buried into the ground. All naturally growing plants within plots were removed when transplanted, and the density of the experimental plants was within the range of the density of the naturally growing plants. Half of each plot (12 plants) was assigned to the insecticide treatment, and the other half was assigned to the control. The position of each plant in the plots was randomly determined. As the pot soil (vermiculite) did not contain nutrients, all plants were supplied with a 1000×diluted solution of Hyponex (N-P-K = 6-10-5, Hyponex, Japan) once a month throughout the experiment.

To manipulate the abundance of insects on the experimental plants, we used a commercially available systemic insecticide, Oltran (Sumitomo Gardening, Japan), which is slow-acting and acephate-based. For each plant assigned to the insecticide treatment, 1 g of Oltran was applied to the surface of the pot soil at the time of transplantation and then every two months during the experiment. The insecticide did not cause any detectable differences in plant morphology.

The number of herbivores on each plant was counted every week in the flowering season, from the end of March to June 2007. To maintain the weak herbivory condition, insects were removed by hand from plants in the insecticide treatment after they were counted. The abundance of herbivores on the control plants was significantly higher than on the insecticide-treated plants (see Results), and thus, the possible influence of the insecticide on the neighboring control plants was considered minimal. We also censused insect herbivores in autumn and winter from the start of the experiment in October 2006 and found that the abundance of herbivores was negligible during this period, even in the control treatment (less than 5% of plants were infested by herbivorous insects). As a measure of female fitness, the number of mature fruits was counted for each plant after the flowering season in June.

In the second experiment to test effect of autumn herbivory alone, another set of 96 plants was transplanted and subjected to natural herbivory under the insecticide and control treatments from 3 October to 27 December 2006. The 96 plants were then transferred again to the laboratory to avoid spring herbivory, because it was difficult to completely exclude insect herbivores in the flowering season and thus the effect of autumn- and spring-herbivory would have been confounded. Plants were prepared and transplanted in the same way as described above, except for the composition of maternal families. In this experiment, four half-sib plants (i.e., two hairy and two glabrous plants from the same mother) were available only for 16 maternal families. The remaining 32 plants consisted of 16 half-sib pairs of hairy and glabrous plants and were assigned randomly to the control or insecticide treatment. Each of the four transplanting plots contained 12 control and 12 insecticide-treated plants.

After three months of growth in the field under autumn herbivory conditions, these 96 plants were brought back to the laboratory and grown for two months at 5°C (10 h light/14 h dark) in the same chamber as described above, followed by flowering at 20°C/10°C (14 h light/10 h dark). Because the species is self-incompatible and flowers were not pollinated in the laboratory, in this case we counted the number of flowers produced instead of fruit production as a measure of plant fitness. In the field, the proportion of flowers that set fruits was high (>80%) unless flowers were consumed by *P. brassicae*. Flower production, therefore, was a good indicator of female fitness under conditions of no floral herbivory.

### Polymorphism of *GL1* and other loci

*GL1* was sequenced for 21 hairy and 22 glabrous plants. The materials sampled were either living plants collected from the field (39 plants), or plants grown in the laboratory that originated from single seeds collected from four maternal plants in the field (four plants). All individuals, including maternal plants from which seeds were collected, were at least 5 m apart to minimize the probability of sampling related individuals, and the most distant plants were separated by 350 m. DNA was extracted from fresh leaves using a DNeasy Plant Minikit (Qiagen) according to the manufacturer's instructions. In a preliminary PCR experiment using primers including those reported in a previous study [37], we could not amplify the entire coding region of the *GL1* gene of glabrous plants. To design new primers, we determined the flanking sequence of *GL1* by thermal asymmetric interlaced (TAIL) PCR [41] and found that the *GL1* gene of glabrous plants contained a large insertion 7 bp upstream of the stop codon in the 3′ end. We designed two pairs of primers to amplify the allele with or without the insertion. Primer pair 1, 5′-TTATAGCCATGATTACACAAAG-3′ (GL1-AF in ref. 37) and 5′-TCGCCCTTTTTAGGAGAGAA-3′, amplified the entire coding region of non-insertion alleles. Primer pair 2, GL1-AF and 5′-TCGAAATTCCGTCGAAAAAC-3′, amplified the entire coding region except for the last 7 bp in the 3′ end, and a part of the insertion sequences of the insertion haplotype. PCR was carried out in 20-µl volumes, with 10 to 20 ng of template DNA, 1×PCR buffer, 0.2 mM dNTPs, 0.25 µM of each primer, and 0.5 units of Ex Taq (Takara Bio, Japan). Cycling conditions were as follows: 94°C (1 min); 30 cycles of 94°C (30 sec), 58°C (30 sec) or 55°C (30 sec) for primer pairs 1 and 2, respectively, and 72°C (1.5 min); and 72°C (3 min). All plants were tested using both primer pairs. For glabrous plants, primer pair 2 amplified *GL1* in all tested plants, whereas no fragment was amplified by primer pair 1. For hairy plants, primer pair 1 amplified *GL1* in all tested plants, and primer pair 2 amplified the insertion haplotype for 12 of 21 individuals examined. PCR products were sequenced directly using a BigDye Terminator Cycle Sequencing Kit version 3.1 and an ABI 3730 DNA Analyzer (Applied Biosystems). Because direct sequencing of the PCR products showed no heterozygous sites, plants were considered heterozygous only when both primer pairs amplified *GL1* haplotypes, and other plants were considered homozygotes. The association of the glabrous phenotype with homozygosity of the insertion allele was perfect, and the *GL1* haplotypes with and without the insertion are referred to as the putative glabrous and hairy haplotypes, respectively. The sequences of 86 haplotypes from the 43 plants were used for polymorphism analysis (see below).

To elucidate the origin of the large insertion in the putative glabrous haplotype, the insertion was sequenced for two glabrous plants. Primer pair 1 was used to amplify *GL1* including the whole insertion by PCR as described above, modified by lengthening the extension reaction to 6 min at 72°C in every cycle. The size of the PCR fragment produced was estimated to be ca. 10 kb. Direct sequencing of the PCR product from both ends by primer walking was unsuccessful because the insertion contained a putative high-dimensional structure and poly-A regions that hindered the sequencing reaction. This region was cloned to facilitate sequencing. The region was amplified by PCR with the same protocol described above, modified by carrying out the extension reaction for 3 min at 72°C in every cycle, and the following primers were used: 5′-CGGTTGACCACTCGCTAGA-3′ and 5′-CGACGGTATTCCGAGAGAGA-3′. The PCR product, which was approximately 4 kb in size, was cloned into a pCR4-TOPO cloning vector using a TOPO TA Cloning Kit for Sequencing (Invitrogen) according to the manufacturer's instructions. Four clones for each individual were sequenced to obtain a consensus sequence. The sequence of a small part of the insertion could not be determined, probably because of the presence of high-dimensional structure. Overall, we determined the sequence of 4578 bp in the 5′ region and 3386 bp in the 3′ region of the insertion. A BLAST search was performed to examine whether the obtained sequence contains previously reported sequences.

To investigate the possibility that other genes linked to *GL1* are associated with trichome variation and that the pattern of polymorphism in *GL1* differs from that of adjacent regions, we also sequenced part of two genes, *DEGP1* (or *AT3G27925*, serine-type endopeptidase) and *AT3G27910* (kelch repeat-containing protein), for the same 43 plants. We chose these genes because they are located adjacent to *GL1* in the genomes of *A. thaliana* and *A. lyrata* (Release Araly1.1, http://www.gramene.org/Arabidopsis_lyrata/Info/Index). Comparative genomics studies of *A. thaliana* (chromosome number n = 5), *A. lyrata* and *A. halleri* (n = 8) showed that chromosome blocks are conserved between these species, while the combination of blocks in a chromosome has changed in the course of chromosome number reduction in *A. thaliana* [42]–[44]. In addition, earlier phylogenetic studies showed that *A. halleri* and *A. lyrata* are more closely related to each other than to *A. thaliana* [45]. Thus, we assumed that the colinearity of the three genes (*DEGP1*-*GL1*-*AT3G27910*) is also conserved in *A. halleri* subsp. *gemmifera*. The intergenic region between *GL1* and *DEGP1* is 3 kb and 6 kb in *A. thaliana* and *A. lyrata*, respectively. *GL1* and *AT3G27910* are separated by 3.8 kb and 12 kb in *A. thaliana* and *A. lyrata*, respectively. The following primers were designed from the conserved regions of the *A. thaliana* and *A. lyrata* genomes and used for PCR: partial sequence (ca. 1.1 kb) of *DEGP1*, 5′-TCCGATCCAAACGCTATTTC-3′, and 5′-TGAAGAGGGGCAAGAGAAAA-3′; partial sequence (ca. 480 bp) of *AT3G27910*, 5′-TTGGGTTGAGGTTTTTGACC-3′ and 5′-TGAATTTTCCAAAGTTAGCACAAG-3′. PCR and sequencing conditions followed those used for the amplification of the *GL1* hairy haplotypes as described above, but the extension time was modified to 1 min in each cycle of PCR. For samples that were heterozygous at a single nucleotide site, two haplotypes with the single nucleotide difference were determined. For samples that were heterozygous at multiple sites and that contained insertion/deletion polymorphisms, PCR products were cloned using a pCR 8/GW/TOPO TA Cloning kit (Invitrogen). Two or more clones were sequenced to obtain a reliable sequence for each haplotype. We determined 86 haplotypes from the 43 plants for both genes assuming no null allele. BLAST searches with the obtained sequences used as queries showed the highest hits to *DEGP1* and *AT3G27910* of *A. thaliana* and clearly distinguished the obtained sequences from other genes. Thus, we could successfully obtain orthologs of these genes in *A. halleri* subsp. *gemmifera*.

We further sequenced exons of five additional genes for 14 hairy and 13 glabrous plants (note that 11 of the 27 plants were not included in the *GL1* sequencing described above). The five genes, *AT1G06520*, *AT2G36980*, *AT3G23590*, *CAF*, and *CHS*, were scattered across the *A. lyrata* genome and chosen arbitrarily from previous population genetics studies on Brassicaceae species [46], [47], wherein the primer sequences and PCR conditions used were described. PCR products, which ranged from ca. 650 to 800 bp in size, were sequenced directly, and the haplotypes of heterozygous individuals were determined using Phase 2.1.1 [48]. All sequences described in this paper have been deposited to GenBank (accession numbers GU071133–GU071162) and DDBJ (AB642298–AB642594).

### Statistical analyses

Statistical analyses for the field census and insect-removal experiment were conducted using R 2.7.1 [49]. The effect of trichome phenotype on fruit production in the field census was examined by fitting generalized linear models (GLMs) with negative binomial error (the glm.nb function implemented in R). The trichome phenotype, rosette diameter (in 2006), and census plot were included as fixed factors. A GLM with negative binomial error was used because many plants produced no fruits, and the frequency distribution of fruit production was strongly overdispersed from the Poisson distribution [50]. The number of *P. brassicae* larvae on plants during the flowering season was examined using a generalized linear mixed effect model (GLMM) with Poisson error (the lmer function) in which the trichome phenotype and plot were included as fixed factors and repeated measurements on each plant as a random factor. AICs (Akaike's information criteria) were compared for models with and without the trichome term. Insects other than *P. brassicae* larvae showed low infestation rates (<2% and <1% of the censused plants infested by adults of *P. brassicae* and larvae of *Pieris napi*, respectively) and were not included in the analyses.

In the insect-removal experiment, the effects of the trichome phenotype and insecticide treatment on fruit production were examined by GLMM with negative binomial error (the negbin function). The trichome phenotype, treatment, rosette diameter, and transplanting plot were included as fixed factors and the maternal plant as a random factor. The number of *P. brassicae* larvae on plants during the flowering season in the transplanting experiment was examined by GLMM with Poisson error in which the trichome phenotype, insecticide treatment, and plot were included as fixed factors and repeated measurements on each individual plant as a random factor. AICs were calculated for the full model and simplified models in which the trichome and treatment terms were sequentially subtracted.

For plants faced only with autumn herbivory, GLM with negative binomial error (the glm.nb function) was fitted to examine the effects of the treatment and trichome phenotype on flower production. Rosette diameter and transplanting plot were also included as covariates.

To investigate how the pattern of *GL1* polymorphism differs from that of the two adjacent genes, Tajima's *D* [51] and Fu & Li's *D* and *F* [52] were calculated for the three genes using DnaSP 4.20 software [53]. Insertion/deletion polymorphisms and the last 7 bp of the coding region downstream of the large insertion in the glabrous haplotype were not included in the analyses. The sequences of *GL1, DEGP1* and *AT3G27910* for *A. lyrata* were obtained from the *A. lyrata* genome database (Release Araly1.1) and used as an outgroup for Fu & Li's *D* and *F* tests.

We also performed a coalescent-based neutrality test for *GL1* using Haploconfig software [54]. In this method, the haplotype frequency distribution, or haplotype configuration, is generated by coalescent simulation under various demographic scenarios, and the observed data are compared with simulated genealogies to test the deviation from neutrality. We employed this haplotype-based test to investigate whether hairy or glabrous haplotype shows a unique pattern of polymorphism [54], [55]. We generated 1,000,000 genealogies by a coalescent simulation conditional on the number of synonymous segregation sites observed. In this test, synonymous insertion/deletion polymorphisms were included as single segregating sites. No recombination was assumed, and the scaled mutation rate θ was derived from the average of the five reference genes. Because the demographic history of the study population is not known, simulations were run under various population history assumptions. In the Haploconfig software, the population size is expressed as follows: given *N* as the present population size, the population size in the past was *N*exp[-β*t*], where *t* is a time unit of *N* generations [54]. In the simulation, the population growth parameter β varied from 0 (constant population size) to 10 (rapid expansion).

## Results

### Field census

The leaf beetle *P. brassicae* attacked the leaves, flowers, flower buds, young fruits and apical meristems of *A. halleri* subsp. *gemmifera* in the flowering season. Compared to plants with an intact apical meristem in the main inflorescence, damage on the apical meristem decreased fruit production by 20.2% and 18.3% in 2005 and 2006, respectively (negative binomial GLM, *P*<0.001 for both years). Nearly half of the plants studied did not produce any fruit (45.2% and 50% in 2005 and 2006, respectively). Thus, herbivory by *P. brassicae* larvae strongly reduced plant fitness by directly damaging reproductive organs.

**Figure 3:**
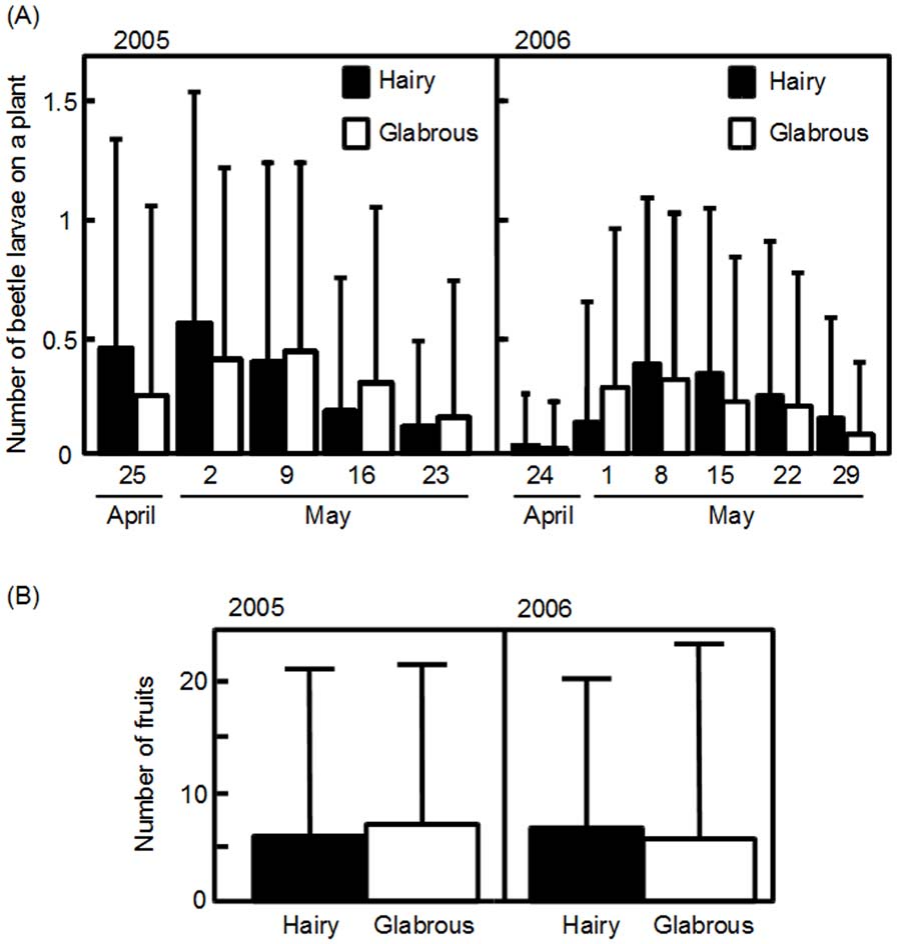
The intensity of herbivory and fruit production in census plots for two years. (A) Mean (+SD) number of *P. brassicae* larvae on hairy and glabrous plants (filled and open bars, respectively) during the flowering seasons of 2005 and 2006 (left and right graphs, respectively). (B) Number of fruits (mean+SD) produced by hairy and glabrous plants (filled and open bars, respectively) for two years. The numbers of hairy and glabrous plants examined were 202 and 262 in 2005 and 160 and 199 in 2006.

We then examined the effect of trichomes on the abundance of *P. brassicae* larvae. The number of *P. brassicae* larvae per plant did not differ between the hairy and glabrous phenotypes throughout the flowering seasons in 2005 and 2006 (Figure 3A). The trichome term in the statistical model did not improve the explanatory power for the number of beetles, as shown by the almost equivalent AICs of the models with and without the term for 2005 and 2006 (Table S1). The presence or absence of damage on apical meristems did not depend on trichome production (Fisher's exact test, *P* = 0.75 in 2005, and *P* = 0.43 in 2006). Furthermore, the level of damage to the leaves also did not differ between hairy and glabrous plants (Figure S1). As expected from the lack of obvious effects of trichomes on herbivory, no significant difference in fruit production was found between hairy and glabrous plants for both years (Figure 3B; Table 1). Plants with a larger rosette size produced more fruits (Table 1), but the rosette size did not differ between hairy and glabrous plants (ANOVA, *P* = 0.49).

**Table 1:** The results of generalized linear models with negative binomial error that evaluated the effect of trichome phenotype on fruit production for 2005 and 2006.

2005	df	Deviance	Residual df	Residual deviance	*P* (χ^2^)
Trichome	1	0.86	463	492.91	0.35
Plot	3	31.78	460	461.13	<0.001
Trichome×Plot	3	6.94	457	454.18	0.07

2006	df	Deviance	Residual df	Residual deviance	*P* (χ^2^)
Trichome	1	1.06	356	598.70	0.30
Rosette size	1	124.68	355	474.02	<0.001
Plot	3	132.32	352	341.70	<0.001

In 2006, rosette size was included in the analysis. As no interaction terms were significant in 2006, the model without interactions is shown.

### Insect-removal experiment

**Figure 4:**
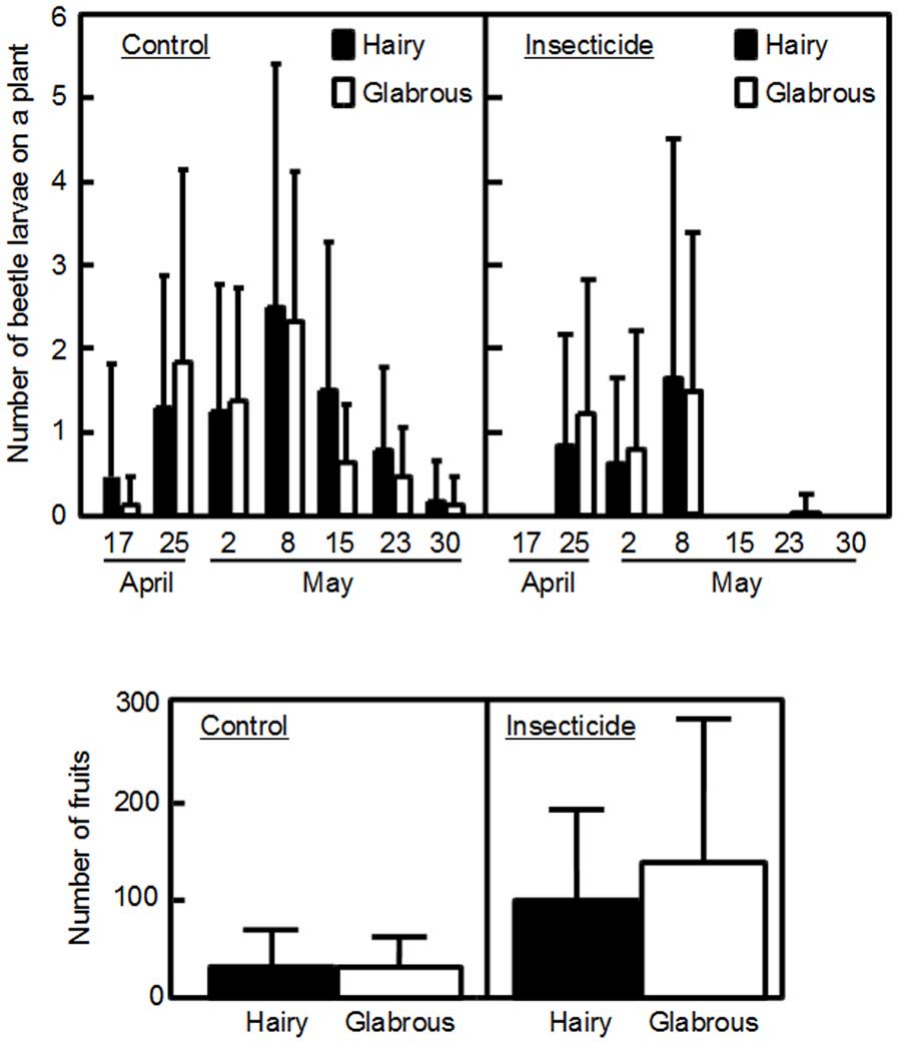
The intensity of herbivory and fruit production in the insect-removal experiment. (A) Mean (+SD) number of *P. brassicae* larvae on hairy and glabrous plants (filled and open bars, respectively) under the control and insecticide treatments (left and right graphs, respectively) in the insect-removal experiment during the flowering season. (B) Mean (+SD) number of fruits produced by hairy and glabrous plants under the control and insecticide treatments (left and right graphs, respectively). The number of experimental plants was 24 for each treatment×trichome combination.

We first examined the number of *P. brassicae* in the transplanting plots. The insecticide treatment greatly reduced the abundance of *P. brassicae* larvae in the flowering season (Figure 4A; Table S2). In both treatments, however, the number of beetle larvae was not different between hairy and glabrous plants during the flowering season (Figure 4A; Table S2). The best model (the lowest AIC model) to explain the number of beetles per plant included the treatment term, but the trichome term did not improve the fit of the model (Table S2).

Next, we examined the effects of the treatment and the trichome phenotype on fruit production. A log-likelihood ratio test showed significant treatment×trichome interaction (the comparison of the first and second models in Table 2, χ^2^ test, *P* = 0.026), which means that the relative fitness of the two phenotypes depended on the experimental treatments. When the transplanted plants were subjected to natural herbivory (the control treatment), the mean fruit production did not differ between hairy and glabrous plants (Figure 4B). The equivalent fitness of hairy and glabrous plants in the control treatment was consistent with the observations made in the census plots described above. In contrast, glabrous plants produced more fruits than did hairy plants in the insecticide treatment (Figure 4B; Table 2). Thus, the cost of trichome production became apparent under the insecticide treatment. Both the number of beetles and fruit production were substantially larger for plants in the insect-removal experiment than in the field census, probably because of the addition of the fertilizer and the mild growth conditions before transplanting.

**Table 2:** AICs of the generalized linear mixed effects models that explain the number of fruits produced in the insect-removal experiments.

Independent variables in models	Term subtracted	AIC
Tre+Tri+(Tre×Tri)+R+P		966
Tre+Tri+R+P	Tre×Tri	974
Tre+R+P	Tri	969
Tri+R+P	Tre	997

The AICs for the models with and without the trichome and insecticide treatment terms were compared. One term was subtracted sequentially from the top model. Abbreviations: Tre, Treatment; Tri, trichome phenotype; R, rosette diameter; P, transplanting plot.

For plants that were exposed only to autumn herbivory, neither the trichome phenotype nor the treatment affected flower production in the laboratory (Figure S2). Thus, autumn herbivory was not a major determinant of plant fitness. The cost of trichome production was not observed in the experiment in which plants were grown in the natural environment for only three months. Therefore, the cost of trichome production appeared to be condition dependent.

### Polymorphism of *GL1* and other loci

Four *GL1* haplotypes were found from 21 hairy and 22 glabrous plants (Table 3). All of the 22 glabrous plants contained only the haplotype with the large insertion (considered the homozygote G1G1), and no polymorphism was found. In contrast, three non-insertion haplotypes (H1, H2 and H3) were found from the 21 hairy plants, and no nonsynonymous polymorphism was found among them. The genotype frequencies of the hairy plants were as follows: 8 plants were H1H1, 1 was H2H2, 9 were H1G1, 2 were H2G1 and 1 was H3G1 (Table 3).

**Table 3:** The haplotype frequency and polymorphic sites of *GL1* for 21 hairy and 22 glabrous plants.

Haplotype	Frequency	Position-45	424	822	941	958	1383
H1	25	TC	G	G	TA	C	C
H2	4	TC	A	T	TA	C	C
H3	1	Deletion	G	G	TA	C	C
G1	56	TC	G	G	Deletion	A	CACTA insertion

Positions of polymorphic sites represent distance from the start codon in the first exon. Polymorphic sites other than the CACTA insertion were located in the 5′ upstream region and the second intron.

**Figure 5:**
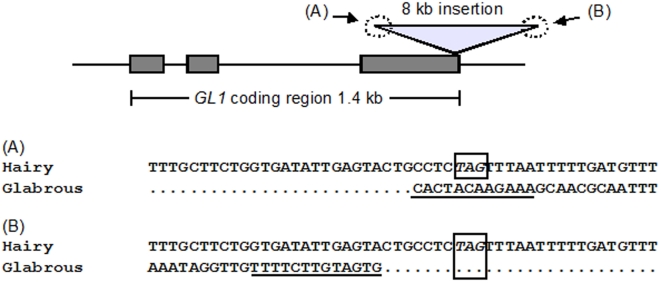
Large insertion in *GL1* of glabrous plants. The structure of *GL1* and sequences of both ends of a long insertion in the third exon in the glabrous haplotype (indicated by (A) and (B)) are shown. Gray boxes represent exons, and the triangle represents the insertion in the putative glabrous haplotype. The corresponding sequence of the haplotype without the insertion (the putative hairy haplotype) is shown for comparison. Nucleotides shown in italics with open boxes indicate a stop codon. Underlined sequences are terminal inverted repeats at both ends of the insertion in the glabrous haplotype, showing typical features of CACTA-family transposons.

The large insertion at the third exon in the putative glabrous haplotype was nearly 8 kb in length and had a terminal inverted sequence in the both ends (Figure 5). This is a typical feature of CACTA-family transposons [56], [57]. The BLAST search also showed that this insertion contained a pseudogenic En/Spm-like DNA transposon. The length of the predicted amino acid sequence was 228 aa for the putative hairy haplotypes, whereas the inferred amino acid sequence of the putative glabrous haplotype contained an additional 62 aa. Other mutations unique to the putative glabrous haplotype were located in the second intron (Table 3).

Two haplotypes containing one single nucleotide polymorphism were found in the partial sequence of *AT3G27910* (458 bp). The partial sequence of *DEGP1* (1.1 kb) contained three haplotypes with 19 segregating sites, including insertion/deletion polymorphisms. These polymorphisms exhibited significant linkage disequilibrium with *GL1* (Table S3, Fisher's exact test, *P*<0.001 for both flanking genes), supporting the assumption that these loci are located in proximity to *GL1* in the *A*. *halleri* genome. However, the polymorphisms found in these two genes were not associated with trichome variation (Table S3). The nucleotide diversity and neutrality test statistics of *GL1* were intermediate or lowest among the three adjacent loci (Table 4). Although Tajima's *D* for silent sites in *GL1* was larger than *DEGP1*, the lack of synonymous polymorphism in *AT3G27910* did not allow the comparison of this statistic for three genes.

**Table 4:** Statistics of diversity and neutrality tests for *GL1* and two genes, *DEGP1* and *AT3G27910*, that are assumed adjacent to *GL1*. For Fu & Li's *D* and *F*, orthologous sequences of *A. lyrata* are used as outgroups.

Statistic	*DEGP1*	*GL1*	*AT3G27910*
π_total_	0.00241	0.00044	0.00089
π_s_	0.00383	0.00069	0
Tajima's *D*_total_	−0.303	0.130	1.237
Tajima's *D*_s_	0.031	0.130	−
Fu & Li's *D*	1.564	0.841	0.498
Fu & Li's *F*	1.104	0.726	0.835

Subscripts indicate estimates based on all sites (total) and synonymous sites (s).

We performed a coalescent simulation to test whether the observed pattern of haplotype frequency in the *GL1* locus was unlikely under neutral evolution. Five reference genes were sequenced to obtain the scaled mutation rate for the coalescent simulation (Table S4). Of the five genes, three showed no polymorphism, and only nonsynonymous polymorphisms were found in the other two genes (Table S4). Because of the lack of synonymous mutations in these five genes, the average mutation parameter was estimated for all sites, θ = 0.2811 (per-locus estimate for *GL1*). As five loci were not sufficient to precisely infer population history, a wide range of demographic assumptions was assumed in coalescent simulations. The haplotype configuration test (Haploconfig software) showed no evidence of selection in any of population history scenarios (Table 5). Because variation in θ affects the results of simulations [54], we performed the same simulation using a prior uniform distribution of θ [0, 5] implemented in Haploconfig and obtained a similar result (Table S5). Thus, our result was robust to uncertainty in population history and the pattern of mutation accumulation.

**Table 5:** Haplotype configuration test of *GL1*.

Population growth	β = 0	β = 0.1	β = 1	β = 2	β = 5	β = 10
Probability	0.829	0.838	0.623	0.385	0.108	0.068

Cumulative probabilities for the observed haplotype configuration are shown. Simulation parameters, sample size = 86; number of synonymous segregation sites = 5; per-locus mutation parameter θ = 0.2811 (derived from the average of five unlinked loci); population growth rate β varied from 0 (constant population size) to 10 (rapid population expansion).

## Discussion

### Is trichome variation adaptive in the study population?

Within-population variation in a defense trait has been found in various plant species [8]–[10]. The coexistence of distinct defense phenotypes within single populations has often been explained by the action of balancing selection. The balancing selection hypothesis states that the defense confers higher fitness in the presence of enemies, that it involves a fitness cost [58]–[61], and that the intensity of herbivory fluctuates in space and time [11], [12]. Genetic variation within the population may also be influenced by other processes such as new mutation, neutral drift and gene flow from nearby populations. In the present study, we found a fitness cost of trichomes in the insect-removal treatment. However, hairy and glabrous plants showed equivalent fitness both in the census plots observed for two years and in the transplanting plots under natural herbivory by *P. brassicae*. The pattern of polymorphism in the candidate gene *GL1* also showed no clear evidence of the long-term maintenance of trichome variation within the study population. We do not exclude the possibility that balancing selection on trichome variation had occurred within the study population. Our results suggest, however, that balancing selection at the single population level, even if it occurred, may not have been a dominant process in shaping trichome variation.

A caveat is that we do not know how non-selective processes such as demographic history and gene flow have affected trichome variation in the study population. Under any assumption on population history, however, the lack of polymorphism in the glabrous allele of *GL1* cannot be explained if balancing selection within the population has played a major role in shaping trichome variation. Even if balancing selection occurred in the past, its influence for increasing polymorphism of *GL1* must have been overwhelmed by other processes that diminished the polymorphism of the glabrous allele.

Our results are in contrast with a study on trichome variation in *A. lyrata* in which a fitness advantage of trichome production was found [27], [28]. However, a number of studies on variation in a defense trait against herbivores and pathogens have shown that the benefit of the trait was not clear, whereas the cost of defense was significant. The cost of trichome production was also found in *A. kamchatica*, in which glabrous plants produced more fruits than did hairy plants [62]. In natural populations of *D. wrightii*, in which strongly defended sticky plants and weakly defended velvety plants coexist, a higher cost of sticky trichomes was found, but there was no evidence for balancing selection acting on the two phenotypes [12], [61]. Similarly, a cost of a resistance gene to a pathogenic infection was found but no significant benefit of the gene was detected in *Ipomoea purpurea* [63]. Recent studies on plant resistance genes have also found patterns of genetic polymorphism that cannot be explained by either positive selection alone or balancing selection alone [15], [64]. Furthermore, a meta-analysis of phenotypic selection studies unveiled the temporary dynamic nature of selection acting in the wild [65]. Thus, the simple form of balancing selection at the single population level may not be a general process for the maintenance of defense variation. For trichome variation in *A*. *halleri* subsp. *gemmifera*, we found that trichomes serve as a defense against larvae of the butterfly *P. napi* (T. Kawagoe & H. Kudoh, unpublished data). The influence of herbivores other than *P*. *brassicae* for plant fitness could not be examined in the study population because of their low abundance. The hairy phenotype may be advantageous in populations which harbor different herbivore communities. If gene flow occurs between local populations, geographic variation in selection on trichome variation can affect the phenotypic frequency of single populations. Thus, it will be valuable to investigate relative effects of local selection, demography and gene flow on trichome variation in a metapopulation framework.

It remains to be answered why hairy and glabrous plants exhibited equivalent fitness under intense herbivory despite the cost of trichome production. The lack of a fitness difference between the two trichome phenotypes may be due to highly intense herbivory. Nearly half of the naturally grown plants did not produce any fruit, and 70% of plants produced five or less fruits. Thus, in terms of fruit production, intense herbivory is likely to hinder the cost of trichome production becoming apparent. Alternatively, the cost of trichome production in hairy plants might be counterbalanced by a small advantage of defense under intense herbivory. This is less likely, however, because the abundance of beetles found on hairy and glabrous plants did not differ significantly.

### *GL1* polymorphism in the natural population

All of the glabrous plants we examined were homozygous for the large insertion that originated from a CACTA-family transposon, while the polymorphisms found in two putative adjacent genes were not associated with trichome phenotypes. Furthermore, these flanking genes are not known to be involved in trichome development in *A. thaliana* [33], [34]. It is less likely that a mutation that disrupts the function of the regulatory region causes the loss of trichomes, because *GL1* was transcribed in both trichome phenotypes (Figure S3). Although we cannot rule out the possibility that mutation in other linked loci is responsible for the glabrous phenotype, our finding of 100% association of the homozygosity of the large insertion with the glabrous phenotype suggests that *GL1* is involved in trichome variation. In *A. thaliana*, the expression of *GL1* in trichome cells in a precise manner requires an enhancer in the 3′ downstream non-coding region [66]. The large insertion in the 3′ end of the glabrous haplotype may inhibit the normal functioning of the enhancer. If the insertion haplotype is involved in the loss of trichomes, our study system may provide insight into the debate about how transposon-induced phenotypic variation contributes to the evolution of host organisms [67]–[75].

### Conclusion

Ecological genomics has begun to clarify the genetic basis of ecologically important traits, such as morphology, defense against herbivores and pathogens, and life history traits [2], [6], [7], [76]–[81]. Molecular evolutionary studies of functional genes have made a great contribution to our understanding of adaptive variation in the last decade [6]. In most cases, however, the ecological consequences of genetic variation in the natural environment still remain unclear (but see [17], [82]). We addressed this issue by combining ecological and candidate gene approaches. Investigation of processes driving the evolution of genetic variation within single populations will be the first step towards thorough understanding of how genetic variation has been shaped at the species level.

## Supporting Information

Figure S1 **The intensity of damage on leaves of hairy and glabrous plants at the end of the flowering seasons for two years.** Trichome phenotype did not affect damage levels (Fisher's exact test, *P* = 0.52 in 2005; *P* = 0.15 in 2006).
(TIF)

Figure S2 **Mean (+SD) number of flowers produced by plants grown for three months in 2006 autumn and allowed to flower in the laboratory.** H, hairy plants; G, glabrous plants. Generalized linear model with negative binomial error: treatment, χ^2^ test, *P* = 0.71; trichome, *P* = 0.768; rosette size, *P*<0.001; block, *P* = 0.9; treatment×trichome, *P* = 0.570.
(TIF)

Figure S3 **RT-PCR to examine** ***GL1*** **expression for four hairy (H) and four glabrous (G) plants.** Actin expression is also shown below *GL1* expression. Three hairy and three glabrous plants showed clear GL1 expression, and one hairy plant (H-4) showed very weak expression. M, molecular marker; “+” and “−”, with and without reverse transcriptase, respectively. RNA was extracted using a Qiagen RNeasy Plant Minikit according to the manufacturer's instructions. cDNA synthesis was performed using 1 µg of RNA and a RETROscript kit (Ambion Inc.). PCR was carried out in 10-µl volumes, with 1 µl of cDNA, 1×Promega GoTaq buffer, 0.2 mM dNTPs, 0.25 µM each primer, and 0.3 µl of self-made Taq polymerase. Cycling conditions were as follows: 94°C (1 min); 35 cycles of 94°C (30 sec), 55°C (30 sec), and 72°C (1.5 min); and 72°C (3 min). Primer sequences were as follows: *GL1*, 5′-CATTATTCGTCTCCACAAGCTCC-3′ and 5′-AGGCAGTACTCAATATCACC-3′; actin, 5′-ATGAAGATTAAGGTCGTGGCA-3′ and 5′-TCCGAGTTTGAAGAGGCTAC-3′.
(TIF)

Table S1 **AICs of the generalized linear mixed effects models that explain the number of leaf beetles during the flowering season in 2005 and 2006.** The AICs for the models with and without the trichome term were compared. Repeated measurements on individual plants were included as a random factor.
(DOC)

Table S2 **AICs of the generalized linear mixed effects models that explain the number of leaf beetles in the insect-removal experiment.** The AICs for the models with and without trichome and insecticide treatment terms trichome term were compared. One term was subtracted sequentially from the top model. Abbreviations: Tre, treatment; Tri, trichome phenotype; P, transplanting plot; Day, the day of census.
(DOC)

Table S3 **Linkage disequilbrium between** ***GL1*** **and two adjacent loci,** ***DEGP1*** **and** ***AT3G27910*** **.** Frequencies of two-locus genotypes are shown.
(DOC)

Table S4 **Diversity of five genes that are assumed unlinked to** ***GL1*** **for 14 hairy and 13 glabrous plants.** *S*, the number of segregating sites; π, nucleotide diversity; θ, the scaled mutation rate per site. Subscripts indicate estimates based on all sites (total) and silent sites (s). For the coalescent simulation, per-locus θ for *GL1* was calculated based on the average of per-site values for the five genes and the length of *GL1* sequence.
(DOC)

Table S5 **Haplotype configuration test (ref. 54 in the main text) for** ***GL1*** **, in which θ was derived from a prior uniform distribution [0, 5].** Other parameters were same as those reported in the main text. Cumulative probabilities for the observed haplotype configuration are shown under various assumptions of population history.
(DOC)
