# Supplementary figures and images for: Coexistence of Trichome Variation in a Natural Plant Population: A Combined Study Using Ecological and Candidate Gene Approaches
Source: PLoS One. 2011 Jul 19;6(7):e22184. doi: 10.1371/journal.pone.0022184 (PMC3139618; doi:10.1371/journal.pone.0022184)

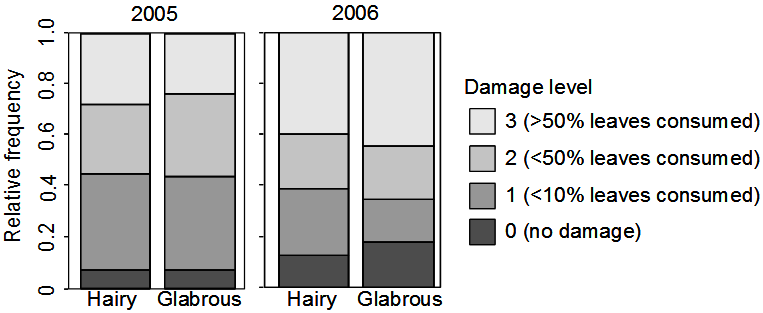

Supplement: Figure S1 — The intensity of damage on leaves of hairy and glabrous plants at the end of the flowering seasons for two years. Trichome phenotype did not affect damage levels (Fisher's exact test, P = 0.52 in 2005; P = 0.15 in 2006). (TIF) [file pone.0022184.s001.tif]

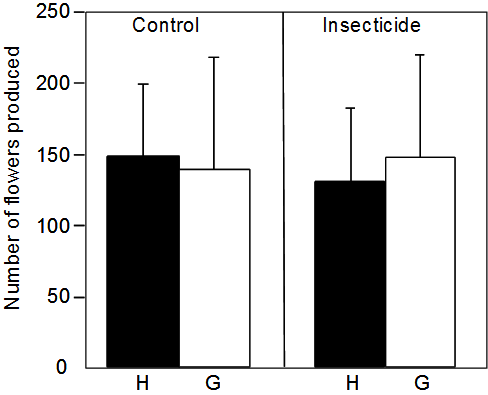

Supplement: Figure S2 — Mean (+SD) number of flowers produced by plants grown for three months in 2006 autumn and allowed to flower in the laboratory. H, hairy plants; G, glabrous plants. Generalized linear model with negative binomial error: treatment, χ2 test, P = 0.71; trichome, P = 0.768; rosette size, P<0.001; block, P = 0.9; treatment×trichome, P = 0.570. (TIF) [file pone.0022184.s002.tif]

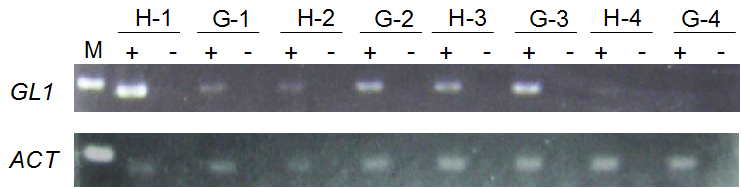

Supplement: Figure S3 — RT-PCR to examine GL1 expression for four hairy (H) and four glabrous (G) plants. Actin expression is also shown below GL1 expression. Three hairy and three glabrous plants showed clear GL1 expression, and one hairy plant (H-4) showed very weak expression. M, molecular marker; “+” and “−”, with and without reverse transcriptase, respectively. RNA was extracted using a Qiagen RNeasy Plant Minikit according to the manufacturer's instructions. cDNA synthesis was performed using 1 µg of RNA and a RETROscript kit (Ambion Inc.). PCR was carried out in 10-µl volumes, with 1 µl of cDNA, 1×Promega GoTaq buffer, 0.2 mM dNTPs, 0.25 µM each primer, and 0.3 µl of self-made Taq polymerase. Cycling conditions were as follows: 94°C (1 min); 35 cycles of 94°C (30 sec), 55°C (30 sec), and 72°C (1.5 min); and 72°C (3 min). Primer sequences were as follows: GL1, 5′-CATTATTCGTCTCCACAAGCTCC-3′ and 5′-AGGCAGTACTCAATATCACC-3′; actin, 5′-ATGAAGATTAAGGTCGTGGCA-3′ and 5′-TCCGAGTTTGAAGAGGCTAC-3′. (TIF) [file pone.0022184.s003.tif]
