# Supplementary material for: Coexistence of Trichome Variation in a Natural Plant Population: A Combined Study Using Ecological and Candidate Gene Approaches
Source: PLoS One. 2011 Jul 19;6(7):e22184. doi: 10.1371/journal.pone.0022184 (PMC3139618; doi:10.1371/journal.pone.0022184)
Supplement: Table S1 — AICs of the generalized linear mixed effects models that explain the number of leaf beetles during the flowering season in 2005 and 2006. The AICs for the models with and without the trichome term were compared. Repeated measurements on individual plants were included as a random factor. (DOC) [file pone.0022184.s004.doc]

**Table S1.** AICs of the generalized linear mixed effects models that explain the number of leaf beetles during the flowering season in 2005 and 2006. The AICs for the models with and without the trichome term were compared. Repeated measurements on individual plants were included as a random factor.

| Independent variables in models | AIC  2005 | 2006 |
| --- | --- | --- |
| Trichome + Plot + Day + (Plot × Day) | 2141 | 1771 |
| Plot + Day + (Plot × Day) | 2142 | 1770 |
