# Supplementary material for: Coexistence of Trichome Variation in a Natural Plant Population: A Combined Study Using Ecological and Candidate Gene Approaches
Source: PLoS One. 2011 Jul 19;6(7):e22184. doi: 10.1371/journal.pone.0022184 (PMC3139618; doi:10.1371/journal.pone.0022184)
Supplement: Table S2 — AICs of the generalized linear mixed effects models that explain the number of leaf beetles in the insect-removal experiment. The AICs for the models with and without trichome and insecticide treatment terms trichome term were compared. One term was subtracted sequentially from the top model. Abbreviations: Tre, treatment; Tri, trichome phenotype; P, transplanting plot; Day, the day of census. (DOC) [file pone.0022184.s005.doc]

**Table S2**. AICs of the generalized linear mixed effects models that explain the number of leaf beetles in the insect-removal experiment. The AICs for the models with and without trichome and insecticide treatment terms trichome term were compared. One term was subtracted sequentially from the top model. Abbreviations: Tre, treatment; Tri, trichome phenotype; P, transplanting plot; Day, the day of census.

| Independent variables in models | Term subtracted | AIC |
| --- | --- | --- |
| Tre + Tri + (Tre × Tri) + P + Day + (P × Day) |  | 1061 |
| Tre + Tri + P + Day + (P × Day) | Tri × Tre | 1059 |
| Tre + P + Day + (P × Day) | Tri | 1057 |
| P + Day + (P × Day) | Tre | 1078 |
