# Supplementary material for: Coexistence of Trichome Variation in a Natural Plant Population: A Combined Study Using Ecological and Candidate Gene Approaches
Source: PLoS One. 2011 Jul 19;6(7):e22184. doi: 10.1371/journal.pone.0022184 (PMC3139618; doi:10.1371/journal.pone.0022184)
Supplement: Table S3 — Linkage disequilbrium between GL1 and two adjacent loci, DEGP1 and AT3G27910 . Frequencies of two-locus genotypes are shown. (DOC) [file pone.0022184.s006.doc]

**Table S3**. Linkage disequilbrium between *GL1* and two adjacent loci, *DEGP1* and *AT3G27910*. Frequencies of two-locus genotypes are shown.

| *GL1* | *DEGP1* *AT3G27910* |
| --- | --- |
|  | AA AB BB AC BC CC CT TT |
| H1H1 4 1 0 1 2 8 0 0  H2H2 1 0 0 0 0 1 0 0  H1G1 0 9 0 0 0 8 1 0  H2G1 0 0 2 0 0 2 0 0  H3G1 0 0 0 0 1 0 1 0  Subtotal for 5 10 2 1 3 19 2 0 hairy plants  G1G1 0 1 21 0 0 4 13 5 (glabrous plants) | |
