# Supplementary material for: Coexistence of Trichome Variation in a Natural Plant Population: A Combined Study Using Ecological and Candidate Gene Approaches
Source: PLoS One. 2011 Jul 19;6(7):e22184. doi: 10.1371/journal.pone.0022184 (PMC3139618; doi:10.1371/journal.pone.0022184)
Supplement: Table S4 — Diversity of five genes that are assumed unlinked to GL1 for 14 hairy and 13 glabrous plants. S, the number of segregating sites; π, nucleotide diversity; θ, the scaled mutation rate per site. Subscripts indicate estimates based on all sites (total) and silent sites (s). For the coalescent simulation, per-locus θ for GL1 was calculated based on the average of per-site values for the five genes and the length of GL1 sequence. (DOC) [file pone.0022184.s007.doc]

**Table S4**. Diversity of five genes that are assumed unlinked to *GL1* for 14 hairy and 13 glabrous plants. *S*, the number of segregating sites; π, nucleotide diversity; θ, the scaled mutation rate per site. Subscripts indicate estimates based on all sites (total) and silent sites (s). For the coalescent simulation, per-locus θ for *GL1* was calculated based on the average of per-site values for the five genes and the length of *GL1* sequence.

| Gene | bp | No. haplotypes | *S*_total_ | *S*_s_ | π_total_ | π_s_ | θ_total_ |
| --- | --- | --- | --- | --- | --- | --- | --- |
| *AT1G06520* | 650 | 1 | 0 | 0 | 0 | 0 | 0 |
| *AT2G36980* | 621 | 1 | 0 | 0 | 0 | 0 | 0 |
| *AT3G23590* | 629 | 3 | 2 | 0 | 0.00073 | 0 | 0.0007 |
| *CAF* | 751 | 1 | 0 | 0 | 0 | 0 | 0 |
| *CHS* | 773 | 2 | 1 | 0 | 0.00037 | 0 | 0.00028 |
