# Supplementary material for: Coexistence of Trichome Variation in a Natural Plant Population: A Combined Study Using Ecological and Candidate Gene Approaches
Source: PLoS One. 2011 Jul 19;6(7):e22184. doi: 10.1371/journal.pone.0022184 (PMC3139618; doi:10.1371/journal.pone.0022184)
Supplement: Table S5 — Haplotype configuration test (ref. 54 in the main text) for GL1 , in which θ was derived from a prior uniform distribution [0, 5]. Other parameters were same as those reported in the main text. Cumulative probabilities for the observed haplotype configuration are shown under various assumptions of population history. (DOC) [file pone.0022184.s008.doc]

**Table S5**. Haplotype configuration test (Innan *et al*. 2005) for *GL1*, in which θ was derived from a prior uniform distribution [0, 5]. Other parameters were same as those reported in the main text. Cummulative probabilities for the observed haplotye configuration are shown under various assumptions of population history.

| Population growth | β = 0 | β = 0.1 | β = 1 | β = 2 | β = 5 | β = 10 |
| --- | --- | --- | --- | --- | --- | --- |
| Probability | 0.752 | 0.730 | 0.499 | 0.3 | 0.203 | 0.088 |
